# Chronisch-obstruktive Lungenerkrankung 2021 – die richtige Therapie für den richtigen Patienten

**DOI:** 10.1007/s00108-021-01021-0

**Published:** 2021-04-16

**Authors:** Horst Olschewski, Roland Buhl, Georg Christian Funk, Arschang Valipour, Claus F. Vogelmeier

**Affiliations:** 1 Klinische Abteilung für Pulmonologie, Universitätsklinik für Innere Medizin, Medizinische Universität Graz, Auenbruggerplatz 15, 8036 Graz, Österreich; 2 Schwerpunkt Pneumologie, III. Medizinische Klinik und Poliklinik, Universitätsmedizin der Johannes Gutenberg-Universität Mainz, Langenbeckstr. 1, 55131 Mainz, Deutschland; 3 2. Medizinische Abteilung mit Pneumologie mit AmbulanzKarl Landsteiner Institut für Lungenforschung und Pneumologische Onkologie, Klinik Ottakring, Montleartstr. 37, 1160 Wien, Österreich; 4 Abteilung für Innere Medizin und Pneumologie, Karl-Landsteiner-Institut für Lungenforschung und Pneumologische Onkologie, Klinik Floridsdorf, Brünner Str. 68, 1210 Wien, Österreich; 5 Klinik für Innere Medizin, Pneumologie, Intensiv- und Schlafmedizin, Universitätsklinikum Gießen und Marburg, Standort Marburg, Baldingerstr. 1, 35033 Marburg, Deutschland

**Keywords:** Inhalative Kortikosteroide, Lang wirksame Muskarinantagonisten, Lang wirksame β‑Mimetika, Asthma bronchiale, Exazerbation, Corticosteroids, inhaled, Muscarinic antagonists, long-acting, Adrenergic beta-agonists, long-acting, Asthma, bronchial, Disease exacerbation

## Abstract

In den vergangenen Jahren wurden auf dem Gebiet der chronisch-obstruktiven Lungenerkrankung (COPD) große Therapiestudien publiziert, die 2020 um mehrere Post-hoc-Analysen ergänzt wurden. Die neuen Erkenntnisse flossen in das Update des Global-Initiative-for-Chronic-Obstructive-Lung-Disease(GOLD)-Report 2021 ein. Im vorliegenden Beitrag werden die aktualisierten Grundlagen und Empfehlungen zur Therapie der COPD beschrieben. Dabei wird auf die Indikationen von inhalativen Kortikosteroiden (ICS), lang wirksamen Muskarinantagonisten (LAMA) und/oder lang wirksamen β‑Mimetika (LABA) eingegangen. Die Therapie der COPD wird der des Asthma bronchiale gegenübergestellt. Diskutiert wird auch, wie sich eine gleichzeitig bestehende Asthmakomponente auf die Behandlungsstrategie bei COPD auswirkt. Ein Schwerpunkt des Beitrags liegt auf der Triple-Therapie mit LAMA, LABA und ICS. In diesem Zusammenhang werden die Studienlage und die Indikationen beschrieben. Die Bronchodilatation bleibt weiterhin die Grundlage der COPD-Therapie. Für Patienten mit gehäuften Exazerbationen bedeutet eine Triple-Therapie mit LAMA + LABA + ICS einen Mortalitätsvorteil. Weitere Analysen oder Studien sollen klären, ob dieser Effekt für spezifische Subgruppen stärker ausgeprägt ist.

## Abstract (en)

In recent years, large treatment studies have been published in the field of chronic obstructive pulmonary disease (COPD) and were supplemented by several post-hoc analyses in 2020. The new evidence was incorporated into the 2021 update of the Global Initiative for Chronic Obstructive Lung Disease (GOLD) report. This article describes the updated fundamentals of and recommendations for the treatment of COPD. Indications for inhaled corticosteroids (ICS), long-acting muscarinic antagonists (LAMA), and/or long-acting beta-mimetics (LABA) will be addressed. The treatment of COPD is contrasted with that of bronchial asthma. The impact of a concomitant asthma component on the treatment strategy for COPD is also discussed. In addition, the article focuses on triple therapy with LAMA, LABA, and ICS, for which the evidence and indications are described. Bronchodilation remains the foundation of COPD therapy. For patients with clustered exacerbations, triple therapy with LAMA + LABA + ICS confers a mortality benefit. Further analysis or studies are needed to clarify whether this effect is more pronounced for specific subgroups.

Das Update des Global-Initiative-for-Chronic-Obstructive-Lung-Disease(GOLD)-Reports 2021 bestätigt die Bronchodilatation als Basistherapie der chronisch-obstruktiven Lungenerkrankung („chronic obstructive lung disease“ [COPD]). Von inhalativen Kortikosteroiden (ICS) bzw. der Triple-Therapie profitieren Patienten mit gehäuften oder schweren Exazerbationen und solche, die gleichzeitig an Asthma bronchiale leiden.

In den vergangenen Jahren wurden auf dem Gebiet der COPD große Therapiestudien publiziert [1–6], die Informationen zum potenziellen Nutzen und Risiko von ICS in Kombination mit einem lang wirksamen Muskarinantagonisten („long-acting muscarinic antagonist“ [LAMA]) und/oder lang wirksamen β‑Mimetikum („long-acting β‑agonist“ [LABA]) liefern. Ergänzend brachte das Jahr 2020 mehrere Post-hoc-Analysen, die die Frage nach einem Mortalitätsvorteil für Patienten mit COPD durch die Gabe von ICS adressierten [7, 8].

Die Erkenntnisse aus diesen Studien und Analysen wurden im GOLD-Report 2021 aufgegriffen [9]. Danach bleibt die Bronchodilatation weiterhin die Grundlage der COPD-Therapie. Für Patienten mit gehäuften Exazerbationen („frequent exacerbators“) bedeutet eine Triple-Therapie mit LAMA + LABA + ICS einen Mortalitätsvorteil. Weitere Analysen oder Studien sollen klären, ob das für spezifische Subgruppen stärker ausgeprägt ist.

## Differenzialdiagnosen zur Exazerbation

Eine Exazerbation ist definiert als eine akute Verschlechterung der respiratorischen Symptome, die zu einer Steigerung der Therapie führt [9]. Doch nicht jede Verschlechterung kommt durch eine Exazerbation zustande. Denken Sie nur an Ihren 70-jährigen Patienten mit Parkinson-Erkrankung, der rezidivierend aspiriert und dadurch wiederholt Pneumonien entwickelt, oder an Ihre 80-jährige Patientin, die immer wieder hypertensive Krisen hat und dann mehr Medikamente braucht. Der GOLD-Report listet eine Vielzahl von Krankheiten als mögliche Differenzialdiagnosen einer Exazerbation auf (Tab. 1). Vielfach unterschätzt wird die akute Lungenembolie, die einer aktuellen Studie zufolge in 6 % der Fälle der respiratorischen Verschlechterung zugrunde liegt [10].

**Table 1:** 

Differenzialdiagnose	Diagnostik
*Pneumonie*	Röntgenuntersuchung des Thorax
	C‑reaktives Protein und/oder Prokalzitonin
*Pneumothorax*	Röntgenuntersuchung des Thorax oder Sonographie
*Pleuraerguss*	Röntgenuntersuchung des Thorax oder Sonographie
*Lungenembolie*	D‑Dimer und/oder Venenduplex
	Computertomographische Pulmonalisangiographie
*Kardiogenes Lungenödem*	Elektrokardiogramm und Echokardiogramm
	Herzenzyme
*Kardiale Arrhythmie – Vorhofflimmern/-flattern*	Elektrokardiogramm

## Bronchodilatation als Basistherapie

Es gibt sehr gute Evidenz, dass eine duale Bronchodilatation mit einem LAMA und LABA im Vergleich zu einer Monotherapie zu einer verbesserten Lungenfunktion und Lebensqualität führt, ohne dass dabei vermehrte Nebenwirkungen auftreten [11]. Es herrschte bisher aber die Meinung vor, das wäre für COPD-Patienten mit hoher Beschwerdelast wichtiger als für Patienten mit geringer Beschwerdelast. Die EMAX-Studie [12] hat bei COPD-Patienten mit einer mittleren Einsekundenkapazität („forced expiratory volume in 1 s“ [FEV_1_]) von 55 % der Norm und COPD-Assessment-Test(CAT)-Scores zwischen 10 und 40 (im Mittel 20) untersucht, wie die Vorteile der dualen Bronchodilatation vs. Monotherapie vom CAT-Score abhängen. Das Besondere der EMAX-Studie war, dass in keinem der Studienarme ICS zum Einsatz kamen. Diese Studie zeigte, dass der Nutzen der dualen Bronchodilatation weitgehend unabhängig vom CAT-Score war und nominell der größte Vorteil im CAT-Bereich zwischen 10 und 20 zu finden war [13]. Das kann als Argument dafür dienen, alle symptomatischen Patienten mit COPD (CAT-Score ≥ 10) von Anfang an mit einer dualen Bronchodilatation zu behandeln.

## Unterschiede zwischen Asthma und chronisch-obstruktiver Lungenerkrankung

**Figure 1:**
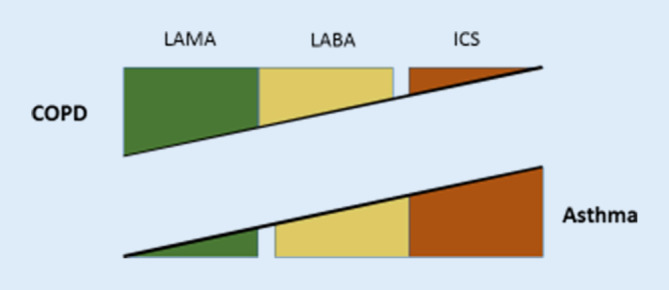

Die Therapie der COPD unterscheidet sich grundsätzlich von jener des Asthma bronchiale. Im Zentrum der COPD-Therapie steht die Bronchodilatation mit einem LAMA, während für das Asthma das ICS die Basis der Therapie darstellt. LABA spielen als Kombinationspartner sowohl bei der COPD als auch beim Asthma eine wichtige Rolle (Abb. 1).

### Triple-Therapie

Sowohl bei der COPD als auch beim Asthma bronchiale wurden in der letzten Zeit Triple-Therapien (LAMA + LABA + ICS) getestet, allerdings unter unterschiedlichen Vorzeichen. Beim Asthma bronchiale verbesserte das zusätzliche LAMA im Wesentlichen die Lungenfunktion [14], bei der COPD verbesserte das zusätzliche ICS die Zahl der Exazerbationen. Es gibt bei COPD auch Hinweise auf einen reduzierten Abfall der FEV_1_ pro Jahr unter inhalativer Therapie, was auf die Effekte von LAMA und ICS zurückzuführen ist [15].

### Praktische Konsequenzen

Unter Berücksichtigung der Ergebnisse der großen Triple-Therapie-Studien [3, 4, 6], der aktuellen Daten der EMAX-Studie [12, 13] und der Interaktion mit dem Zigarettenrauchen [16] ergeben sich in Übereinstimmung mit dem GOLD-Report 2021 [9] für COPD (a) und dem Global-Initiative-for-Asthma(GINA)-Update 2020 [17] für Asthma (b) folgende Differenzialindikationen:

- LAMA-Monotherapie:
  - Für oligosymptomatische COPD-Patienten (CAT-Score < 10)
  - Keine Indikation für Asthma bronchiale
- LAMA + LABA:
  - Für alle symptomatischen COPD-Patienten (CAT-Score ≥ 10)
  - Keine Indikation für Asthma bronchiale
- ICS-Monotherapie:
  - Keine Indikation für COPD
  - Für Patienten mit Asthma bronchiale, die damit eine kontrollierte Erkrankung erreichen
- ICS + LABA:
  - Für oligosymptomatische COPD-Patienten mit vermehrten Exazerbationen, insbesondere wenn folgende Randbedingungen vorliegen: > 300 Eosinophile/µl Blut, Asthmaanamnese oder begleitendes Asthma
  - Für Patienten mit Asthma, die unter ICS-Monotherapie keine kontrollierte Erkrankung erreichen, oder in Form von ICS + Formoterol als Bedarfstherapie in den Stufen GINA 1 und 2
- ICS + LABA + LAMA:
  - Für symptomatische COPD-Patienten mit vermehrten Exazerbationen trotz einer Therapie mit ICS + LABA oder LABA + LAMA
  - Für Patienten mit Asthma, die unter ICS + LABA keine Asthmakontrolle erreichen

**Figure 2:**
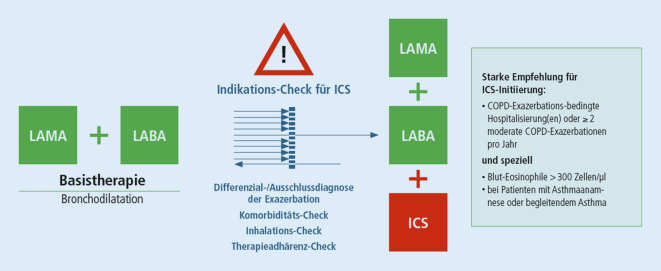

In Abb. 2 sind Überlegungen zusammengefasst, die zu Beginn einer ICS-Therapie angestellt werden sollten [9, 18].

## Senkung der Mortalität von COPD-Patienten durch Triple-Therapie – Mythos oder Realität?

Die 3‑armige IMPACT-Studie (*n* = 10.355) untersuchte COPD-Patienten mit einer hohen Exazerbationsrate und randomisierte sie auf LABA + LAMA vs. ICS + LABA vs. ICS + LABA +LAMA. Der primäre Endpunkt war die Exazerbationsrate. Die Mortalität im Triple-Arm (und im LABA/ICS-Arm) war signifikant niedriger als im LABA/LAMA-Arm [4]. Die Mortalität war allerdings nicht der primäre Endpunkt der Studie. Eine Post-hoc-Analyse, in der alle Mortalitätsereignisse erfasst und analysiert wurden, bestätigte die erniedrigte Mortalität im Triple-Therapie-Arm [7]. Allerdings war der beobachtete Effekt auf Patienten beschränkt, die bereits vor Einschluss in die Studie eine ICS-haltige Therapie erhalten hatten (s. unten).

Die 4‑armige ETHOS-Studie (*n* = 8509) untersuchte die Exazerbationsrate bei COPD-Patienten mit einer hohen Exazerbationsrate (ICS + LABA vs. LABA + LAMA vs. niedrig dosiertes ICS + LABA + LAMA vs. hoch dosiertes ICS + LABA + LAMA). Der Therapiearm mit der höheren ICS-Dosis (*n* = 2137) in der Triple-Kombination zeigte im Vergleich mit der LABA + LAMA-Kombination eine geringere Mortalität [6]. Hier gab es keinen Unterschied zu LABA + ICS, es machte aber auch keinen Unterschied, ob die Patienten vor Studienbeginn eine ICS-haltige Therapie erhalten hatten oder nicht [8].

Umstritten ist bislang, welche Rolle das Absetzen von ICS in der Gruppe von Patienten gespielt haben könnte, die in den Studien mit LABA + LAMA behandelt wurden. So haben Suissa u. Ariel [19] eine Analyse der monatlichen Exazerbationsraten in IMPACT und TRIBUTE vorgelegt, wonach Unterschiede zwischen dem Triple-Arm und dem LABA + LAMA-Arm nur in den ersten Monaten zu beobachten waren. Diese Interpretation der Befunde wurde kontrovers diskutiert [20].

Dennoch legen die mit vergleichbaren Patientengruppen und unterschiedlichen Substanzen erzielten Ergebnisse nahe, dass eine inhalative Triple-Therapie gegenüber dualer Bronchodilatation in erster Linie bei symptomatischen Patienten mit einer Anamnese für häufige und/oder schwere Exazerbationen einen günstigen Effekt auf die Mortalität haben könnte.

### Details und mögliche Mechanismen

In die IMPACT-Studie konnten COPD-Patienten mit einer Asthmaanamnese eingeschlossen werden; Patienten mit deutlich eingeschränkter Lungenfunktion und sehr hohem Exazerbationsrisiko waren überrepräsentiert. Der abrupte Entzug von ICS bei Randomisierung in den LABA + LAMA-Studienarm kann gerade bei Patienten mit (subklinischer) asthmatischer Komponente trotz klinisch führender COPD die Studienergebnisse maßgeblich beeinflussen [19, 21]. Diese Annahme wird im Umkehrschluss durch Ergebnisse der IMPACT-Studie unterstützt, die keinen Nutzen einer ICS-Therapie bei Patienten zeigte, die vor Studieneinschluss keine ICS-Therapie hatten. In dieser Subgruppe war die Mortalität unter Triple-Therapie vergleichbar mit der Mortalität unter einer dualen Bronchodilatation (Hazard Ratio 1,25; 95 %-Konfidenzintervall [KI] 0,60–2,59), in deutlichem Unterschied zu Patienten mit einer ICS-Therapie vor Studieneinschluss (Hazard Ratio 0,63; 95 %-KI 0,44–0,89).

Das abrupte Absetzen von ICS vor Studienbeginn kann die Studienergebnisse maßgeblich beeinflussen

Die niedrige Mortalität unter Triple-Therapie bei Patienten mit einer ICS-Vortherapie im Kontrast zum frühen und deutlichen Mortalitätssignal bei Randomisierung im LAMA + LABA-Studienarm passt zu der Annahme, dass es der Entzug der ICS-Therapie ist, der die Mortalität entscheidend beeinflusst. Dazu passt auch die nahezu identische Mortalität in dieser Population in den Triple- und ICS + LABA-Studienarmen [7, 22, 23].

### Rolle eines subklinischen Asthma bronchiale

Es ist seit Langem bekannt, dass bei Patienten mit (subklinischer) asthmatischer Komponente trotz klinisch führender COPD, also bei Patienten mit einer anamnestischen Asthmaerkrankung und/oder asthmatypischem Symptomprofil (u. a. hohe Exazerbationsfrequenz, erhöhte Eosinophilenzahlen im peripheren Blut, bronchiale Hyperreagibilität; [24, 25]) und ICS-Therapie durch vorbehandelnde Ärzte, ein abrupter ICS-Entzug einen in doppelter Hinsicht negativen Einfluss auf die Mortalität hat. So erhöht die Beendigung einer ICS-Therapie bei Patienten mit Asthma die Mortalität innerhalb von nur 3 Monaten um mehr als den Faktor 4 („rate ratio“ 4,6; 95 %-KI 1,1–19,1; [19, 21, 26]). Umgekehrt führt eine Behandlung von Asthmapatienten nur mit einem LABA in gleicher Weise zu einer erhöhten Mortalität (Serevent Nationwide Surveillance Study: „rate ratio“ 3,0; 95 %-KI 0,7–20,0; Salmeterol Multicenter Asthma Research Trial: „rate ratio“ 4,4; 95 %-KI 1,2–15,3; [27, 28]). Angesichts der Tatsache, dass bei 70 % der in der IMPACT-Studie in den LAMA/LABA-Arm randomisierten Patienten eine ICS-Vortherapie plötzlich beendet wurde, erscheinen diese Punkte umso relevanter.

## Typ-2-Signatur oder eosinophile COPD?

Asthma und COPD sind Erkrankungen mit unterschiedlichen Ätiologien, die allerdings gleichzeitig bei einem Patienten vorkommen können. Patienten mit COPD und gleichzeitigem Asthma haben eine deutlich höhere Exazerbationsfrequenz und deutlich schwerere Exazerbationen [29]. Etwa 20 % der COPD-Patienten haben zusätzlich eine typische Asthmacharakteristik bzw. eine Typ-2-Signatur [30, 31]. Dafür sprechen erhöhte Eosinophilenzahlen im Blut und im Sputum und erhöhte Werte der Fraktion des exhalierten Stickstoffmonoxids (FeNO) sowie eine allergische Diathese des Patienten oder seiner Familie. Auch eine subklinische Typ-2-Signatur kann sich in der Exazerbation demaskieren. So wiesen 28 % der Patienten mit COPD-Exazerbation im Sputum und im Blut Charakteristika von Patienten mit Asthmaexazerbation auf [32]. Ob auch die bronchiale Hyperreagibilität für die Differenzialindikation eine Bedeutung hat, kann nicht abschließend beurteilt werden.

## Rolle des Zigarettenrauchens

Das Rauchen spielt sowohl für das Asthma als auch für die COPD eine wichtige Rolle: Der Schweregrad eines Asthmas kann durch Rauchen gesteigert werden, bei gleichzeitig vermindertem Ansprechen auf eine ICS-Therapie. Es kommt bei Rauchexposition zu einer stärkeren Obstruktion und mehr Exazerbationen [33]. Die COPD ist neben den Gefäßerkrankungen und Lungenkrebs bekanntermaßen die häufigste Folgekrankheit des Rauchens. Durch das Beenden des Rauchens verlangsamt sich der jährliche Abfall der Lungenfunktion. Bei Asthma führt das Zigarettenrauchen früher zu einer fixierten Obstruktion als bei Patienten ohne Asthma. Daher weisen die Asthmatiker unter den COPD-Patienten besonders wenig Packungsjahre auf. Umgekehrt ist bei COPD-Patienten kein Ansprechen auf ICS nachweisbar, wenn < 2,4 % Eosinophile und > 46 Packungsjahre vorliegen [16]. Das bedeutet, dass auch eine niedrige Zahl von Packungsjahren beim COPD-Patienten einen wichtigen Hinweis auf eine Asthmakomponente darstellen kann.

In Summe machen diese Argumente deutlich, dass die Mortalitätssenkung im Zusammenhang mit einer Triple-Therapie bei COPD vermutlich auf Patienten beschränkt ist, die bestimmte Voraussetzungen mitbringen. Dazu zählen Patienten mit häufigen und/oder schweren Exazerbationen unter schon bestehender Therapie in Verbindung mit erhöhten Eosinophilenzahlen im peripheren Blut. Empfehlungen der GOLD und European Respiratory Society (ERS) ebenso wie aktuelle Metaanalysen stützen diese Annahme [34–37]. Die Bedeutung einer möglichen Asthmakomponente für diese Konstellation bleibt zu klären.

## Fazit für die Praxis

- Die Bronchodilatation bleibt die Grundlage der Therapie bei chronisch-obstruktiver Lungenerkrankung (COPD).
- Die wissenschaftliche Evidenz unterstützt die Empfehlung, die Gabe von Kombinationstherapien mit inhalativen Kortikosteroiden den Patienten mit COPD und vermehrten Exazerbationen vorzubehalten. Abgesehen von häufigen und/oder schweren Exazerbationen sind die Patienten klinisch charakterisiert durch erhöhte Eosinophilenzahlen im Blut.
- Eine Exazerbation ist definiert als eine akute Verschlechterung der respiratorischen Symptome, die zu einer Steigerung der Therapie führt. Doch nicht jede Verschlechterung kommt durch eine Exazerbation zustande.
- Eine niedrige Zahl von Packungsjahren beim COPD-Patienten kann ein wichtiger Hinweis auf eine Asthmakomponente sein.
